# Diet-Induced Obesity and Circadian Disruption of Feeding Behavior

**DOI:** 10.3389/fnins.2017.00023

**Published:** 2017-02-07

**Authors:** Aurea Blancas-Velazquez, Jorge Mendoza, Alexandra N. Garcia, Susanne E. la Fleur

**Affiliations:** ^1^Institute of Cellular and Integrative Neurosciences, Centre National de la Recherche Scientifique UPR-3212, University of StrasbourgStrasbourg, France; ^2^Department of Endocrinology and Metabolism, Academic Medical Center, University of AmsterdamAmsterdam, Netherlands; ^3^Metabolism and Reward Group, Netherlands Institute for NeuroscienceAmsterdam, Netherlands

**Keywords:** circadian, hypothalamus, reward, feeding, obesity, clock-genes, palatable, dopamine

## Abstract

Feeding behavior shows a rhythmic daily pattern, which in nocturnal rodents is observed mainly during the dark period. This rhythmicity is under the influence of the hypothalamic suprachiasmatic nucleus (SCN), the main biological clock. Nevertheless, various studies have shown that in rodent models of obesity, using high-energy diets, the general locomotor activity and feeding rhythms can be disrupted. Here, we review the data on the effects of diet-induced obesity (DIO) on locomotor activity and feeding patterns, as well as the effect on the brain sites within the neural circuitry involved in metabolic and rewarding feeding behavior. In general, DIO may alter locomotor activity by decreasing total activity. On the other hand, DIO largely alters eating patterns, producing increased overall ingestion and number of eating bouts that can extend to the resting period. Furthermore, within the hypothalamic areas, little effect has been reported on the molecular circadian mechanism in DIO animals with *ad libitum* hypercaloric diets and little or no data exist so far on its effects on the reward system areas. We further discuss the possibility of an uncoupling of metabolic and reward systems in DIO and highlight a gap of circadian and metabolic research that may help to better understand the implications of obesity.

## Introduction

Biological rhythms are the cyclic variations of any biological process of a living organism. Rhythms with a ~24 h duration are called circadian, a word with Latin etymologies that means *circa* (around) and *dies* (day). Circadian rhythms are adaptive to the cyclic environment caused by the rotation of the earth on its own axis, where the most evident variation is the light-dark (LD) cycle, causing the day and night. General locomotor activity and food intake are two of the behavioral outputs of the endogenous circadian system which, in normal feeding and LD conditions, are coupled and synchronized to the activity period of the organism; predominantly during the day in humans and during the night in most rodents (Aschoff, 1981; Silver and LeSauter, 2008). Biological rhythms are not only displayed as a response to the environmental changes but they are inherently paced by a timekeeping system comprised of several organs, tissues, and brain nuclei called oscillators. The rhythmic properties of these oscillators can be observed, for instance, in the electrical activity of the cells, neurotransmitter and molecule synthesis and release, or gene expression. In natural conditions, these oscillators can be entrained by several external or environmental factors (such as the alternance of day-night, food availability, and/or temperature) that set the timing of their functions (Rensing and Ruoff, 2002; Challet, 2010). The main synchronizer or *zeitgeber* (ZT; a German noun adopted to define a time-giver) is the solar time, which is able to pace the activity/inactivity cycles (in chronobiology ZT0 is used to indicate the start of the light period). Thus, activity cycles are entrained by photic signals that are received by the ganglion cells in the retina and transmitted via the optic tract to the hypothalamic suprachiasmatic nuclei (SCN; Albrecht, 2012). These nuclei, located bilaterally adjacent to the third ventricle and dorsal to the optical chiasm, are considered the main biological clock since its physical or genetic function ablation causes disorganized locomotor activity as well as disrupted eating and drinking rhythmic patterns (Stephan and Zucker, 1972; Albus et al., 2002).

Food ingestion, an essential part of energy balance, is controlled by two main processes in the brain: One that evaluates the quantity of the required energy intake and another that regulates the quality of the food including its hedonic properties (Berthoud and Morrison, 2008). The first system, in charge of the energy balance, resides in the hypothalamus, a central area that receives and sends information from and to the peripheral organs via neuronal and hormonal signals (Schwartz et al., 2000; Lenard and Berthoud, 2009). Whereas, the reward-limbic system processes the characteristics and quality of the food, reinforcing the preference for palatable/rewarding items, which in general contain high levels of sugar and/or fat (Avena et al., 2012). Nowadays, people have a large choice of food items, due to the relatively easy access to ultra-processed food. The exposure to food-enriched environment, together with social variables like education and socioeconomical status, determine the food choice made by an individual (Drewnowski and Specter, 2004). The obesity epidemic and the concomitant metabolic diseases are thus, partly caused by the overexposure to palatable food choices (Juul and Hemmingsson, 2015; Louzada et al., 2015). But the caloric consumption beyond homeostatic need does not fit into the energy homeostasis model. One hypothesis is that the exposure to an enriched food environment stimulates our visual, olfactory and gustatory senses, overriding the energy-balance system by means of over excitation of the reward system (Zheng et al., 2009). In obese humans, behavioral changes such as an increase in depressive symptoms and disruption of sleep patterns indicates a close relationship between the reward and circadian systems (Kudlow et al., 2013; Ulrich-lai et al., 2016). Moreover, the relationship between food intake and the circadian system is observed in people that are night-workers who are forced to change their normal activity rhythms which is associated with increased risk to develop obesity and metabolic diseases (Peplonska et al., 2015). Another situation that links circadian rhythms and food intake is the night eating syndrome in which people binge on food during the normal resting period consuming a big amount of calories coming from hypercaloric palatable foods (Gallant et al., 2012).

This review focuses on the data available from mice and rat models aiming to determine the effects of hyper caloric diets on the daily rhythms of locomotor activity and feeding behavior. Furthermore, we describe the current evidence how hyper caloric diets affect circadian properties of the homeostatic and reward systems.

## Cycles of the homeostatic hypothalamic clock(s)

The hypothalamus is a brain center that integrates internal and external signals to produce vital behaviors such as eating. Within this region, several nuclei important for homeostatic regulation have been identified, including the SCN, the hypothalamic arcuate nuclei (ARC), ventromedial and dorsomedial hypothalamic nuclei (VMH and DMH), paraventricular nuclei (PVN), and lateral hypothalamic area (LH) (Schwartz et al., 2000; Gonnissen et al., 2013). These nuclei contain different cell populations that synthesize and release neuropeptides and neurotransmitters that are important for regulating food intake. Given the vital function of food ingestion, it is not surprising that redundant systems exist within the hypothalamus to ensure this important behavior continues. The presence of several orexigenic molecules in different areas such as neuropeptide Y (NPY) and agouti related peptide (AgRP) in the ARC and the melanin-concentrating hormone (MCH) and the hypocretines/orexin in the LH are evidence of the complex regulation of feeding. In the ARC two anorexigenic peptides pro-opiomelanocortins (POMC) and cocaine and amphetamine regulated transcript (CART) produce signals of satiety (Schwartz et al., 2000). The decrease of circulating glucose and nutrients as well as other hunger signals such as an empty stomach are sensed and processed by the brain to trigger feeding behavior (Berthoud and Morrison, 2008). In conditions where the organism ingests a nutritionally balanced diet (i.e., a laboratory chow diet) feeding behavior is arranged in a temporal manner, which is coupled with the activity period. Although the SCN is considered to be the main biological clock, there are also several organs and brain areas containing oscillatory properties and thus, they are known as peripheral circadian oscillators (Albrecht, 2012). In normal healthy conditions, these peripheral oscillators show different rhythmic features such as a variation in electrical activity, neurotransmitter release, and/or gene expression.

### Neuronal activity

The SCN cells show clear rhythmic electrical activity. In brain slices of hamsters, rats, and mice, electrophysiological experiments have revealed a rhythmic firing rate with higher levels during the light period (Gillette and Reppert, 1987; Shibata and Moore, 1988; Albus et al., 2002). Similarly, the ARC shows clear circadian firing rate even in the absence of the SCN input as evidenced with *ex-vivo* slice preparations (Guilding et al., 2009). In the DMH, although circadian rhythms are present, the amplitude of the oscillations is decreased and dampens faster than those observed in the ARC, whereas the VMH has no clear oscillation after tissue culture (Guilding et al., 2009). Nevertheless, another study in rats, recorded the electrical activity of the VMH *in-vivo*, finding a rhythmic activity with an acrophase (highest activity around the 24 h) in the dark period. Moreover, when the SCN was lesioned, the *in-vivo* rhythmicity of the VMH was blunted (Inouye, 1983). The fact that some nuclei show self-sustained rhythms (although in a lesser extent compared to the SCN), and some others are not able to oscillate in the absence of SCN signals is evidence of the hierarchic nature of the circadian system.

### Diurnal variation of neurotransmitter levels

Neuropeptides and neurotransmitters within the hypothalamus also show circadian rhythmicity. These oscillations can be observed in neuropeptides such as NPY, which has receptors located within hypothalamic nuclei, forebrain and cortex (Keen-rhinehart et al., 2010; Kash et al., 2015). In rats, the expression of both NPY and its receptor Y1R in the ARC and PVN are higher during the active period (Cohen et al., 2015). Interestingly, the NPY and its Y1R receptor were detected in the hippocampus and the basolateral amygdala but no clear day and night difference was observed (Cohen et al., 2015). In mice, gene expression of the AgRP, NPY (Stütz et al., 2007) and orexin (Stütz et al., 2007; Opperhuizen et al., 2016), molecules that increase appetite and decrease metabolism and energy expenditure, exhibit diurnal variation. Moreover, a clear time difference was also seen for MCH and the leptin receptor but not in the levels of POMC or CART (Stütz et al., 2007). A study using immunohistochemistry reported no day-night variation of orexin peptide in the hypothalamus of mice, however, c-fos co-localization with ORX cells was higher during the active period (Marston et al., 2008). Taken together, the evidence suggests that not all hypothalamic molecules implicated in the regulation of feeding and energy balance have the ability to oscillate. The contribution of a single variable in rhythmic behavior was assessed by interfereing with the function of the NPY receptor in the mediobasal hypothalamus. The ablation of NPY signaling in this hypothalamic area produced disturbed sleeping and feeding patterns; i.e., rats increased feeding during the light period (Wiater et al., 2011). Another approach was the use of viral gene transfer to overexpress NPY peptide in the LH and PVN. The overexpression of NPY in the LH but not in the PVN resulted in a reduced amplitude of locomotor activity and disruption of the diurnal eating pattern (Tiesjema et al., 2007). Thus, some alterations can be observed when the NPY rhythmicity is disturbed by constantly suppressing or over-expressing NPY. These studies demonstrate that disrupting the rhythm of one single molecule already disrupt a circadian behavioral rhythm. Further investigation is needed to understand why some molecules have a circadian variation and what implications these oscillations have on physiology.

### Rhythmic clock-gene expression

The molecular gene machinery can be observed in cells throughout the body and is comprised of several molecules that generate rhythms of transduction/translation with a ~24 h duration. In this oscillating mechanism *Bmal1* and *Clock* genes form part of the positive loop, which promotes the transcription of the Period (Per 1–3) and Cryptochrome (Cry 1–2) genes. The latter genes form the negative loop components, which in turn suppresses the activity of *Bmal1* and *Clock* dimer (Takahashi, 2015). These genes and their products are relevant molecules for building the circadian variations in physiology and behavior. *Per1* and *Per2* clock-proteins have been evaluated with immunohistochemistry in both the rat and mouse hypothalami at different time points of the day. A daily rhythmic expression of these proteins is found in the ARC, DMH, and VMH with higher levels at night (Verwey et al., 2007; Feillet et al., 2008). Another way of analyzing PER2 expression is with the *ex-vivo* bioluminescence technique, where the PER2 protein is coupled to a luciferase reporter, which allows the measurement of the photon emission produced when PER2 protein is expressed. Using this method, PER2 in the ARC and DMH of animals kept in light/dark (LD) and dark/dark (DD) conditions has been shown to oscillate even when these nuclei are isolated from the rest of the brain (Guilding et al., 2009; Hughes et al., 2011). Similar results have been found for PER1 bioluminescence in the PVN and LH with the acrophase during the night time (Abe et al., 2002). This technique has made it possible to describe rhythmic properties of clock-genes and their proteins in different brain areas. Moreover, the rhythm in the SCN is stronger than those of the peripheral oscillators which, when isolated, show lower amplitude and their oscillations dampen with a faster rate (Abe et al., 2002). As for the rhythmic expression of clock genes, the electrical activity varies in intensity depending on the brain nuclei (Guilding et al., 2009), but its functionality is not fully understood. Several experiments have demonstrated that the complete knockdown of clock-genes in the body alters physiology and behavior. For instance, the mutation of the *clock* gene includes loss of normal locomotor activity patterns as well as metabolic alterations and obesity (Rudic et al., 2004; Turek et al., 2005). The knockout of systemic *Per2* produces a phenotype that displays disrupted rhythmicity of locomotor activity in constant darkness (DD) conditions, as well as lower body weight and disrupted lipid metabolism (Zheng et al., 1999; Grimaldi et al., 2010). Although there is a large body of evidence that links the clock gene function to metabolic physiology, the implications of the rhythmic clock-gene expression within the different areas of the hypothalamus are still unclear. An attempt to determine the effect of a single clock-gene in a specific area was made within the hypothalamic tuberomammilar nucleus (TMN), an area that integrates inputs from the circadian and the sensory stimuli to modulate locomotion and arousal (Torrealba et al., 2012). When *Bmal1* is knocked down from the histaminergic cells in the TMN of mice their normal rhythmic levels of histamine are lost and they display fragmented sleep no changes in overall locomotor activity (Yu et al., 2014). This highlights the important role a single clock-gene can play in specific brain areas and functions. These findings lead to generate questions about which functions are controlled by each clock-gene in different brain areas.

## The cycles of the reward system

The reward system comprises several nuclei as well as different neurotransmitters, with the mesolimbic dopaminergic pathway playing a central role. This circuit includes the ventral tegmental area (VTA), a nucleus containing dopaminergic cells that projects to the nucleus accumbens (NAc) and the cortex (Koob and Volkow, 2010). Other nuclei that send projections to the NAc are the amygdala and hippocampus, which are involved in the regulation of emotion and memory consolidation (Sesack and Grace, 2010). The bed nucleus of the stria terminalis (BNST) is known to densely project to the amygdala and is considered part of the extended amygdala, where the corticotropin-releasing factor, a neuropeptide involved in stress response, is a key signal (Kash et al., 2015; Daniel and Rainnie, 2016). The septum forms part of the limbic system, where it receives dopaminergic projections from the VTA and regulates affective behaviors (Mogenson et al., 1980; Sokolowski and Corbin, 2012). Beyond the classical mesolimbic dopaminergic circuitry, other structures integrate the wiring of the reward process such as the lateral hypothalamus (LH) and the habenula. The LH is part of the feeding behavior neurocircuitry and it is anatomically connected to the dopaminergic system which regulates motivation and arousal (Harris et al., 2005; Berthoud and Münzberg, 2011; Stuber and Wise, 2016). The habenula is an epithalamic area that can be divided into different sub regions: the medial and lateral part. The medial habenula (MHb) receives inhibitory and excitatory projections from the septum (Qin and Luo, 2009). The lateral habenula (LHb) sends excitatory glutamatergic projections to the tail of the VTA (Matsumoto and Hikosaka, 2007; Bianco and Wilson, 2009). Thus, the LHb is able to modulate dopamine release and it is involved in the processing of the reward-prediction error and negative motivational states (Proulx et al., 2014; Salaberry and Mendoza, 2016). The reward system is known to regulate the appetitive state of the organism by influencing the approach and intake of food. Recently, the function of the LH and the LHb have been shown to communicate and to modulate the control of motivational feeding (Stamatakis et al., 2016). Areas of the reward system have different cyclic properties that can be observed in neuronal activity, neurotransmitter release and gene expression.

### Neuronal activity

By using the neuronal activity marker c-fos, an immediate early gene, Baltazar et al. (2013) found that there is a day-night rhythm of neuronal activity in the rat PFC, NAc, and VTA, with higher levels during the night (Baltazar et al., 2013). A diurnal variation of c-Fos has also been observed in the LH and LHb, with the LH having higher levels during the night in mice (Marston et al., 2008) and the LHb during the daytime around ZT6 in rats (Chastrette et al., 1991; Xu et al., 2015). However, other studies have found in hamsters and also in mice higher levels of c-fos during the night time (2 h after lights off), compared to day time (2 h before lights off; Tavakoli-Nezhad, 2005; Tavakoli-Nezhad and Schwartz, 2006). The different results found for c-fos could be due to species differences and/or the time point of when the c-fos was evaluated. Despite these discrepancies, the findings point to the existence of rhythmic c-fos expression in the LHb. Another piece of evidence of direct neuronal activity comes from experiments using cellular multi-unit recordings, where a cyclic variation of electrical activity in the NAc and medial septum of hamsters has been measured *in-vivo* (Yamazaki et al., 1998). This observation has also been reported *in vitro* with LHb cells of mice, where the firing rate was found to peak during the latter part of the light period (Sakhi et al., 2014). In rats this activity has been evaluated in *in vivo* and *in vitro* with both showing the highest activity around ZT6 (Zhao and Rusak, 2005).

### Diurnal variation of neurotransmitter levels

As stated previously, the neuronal activity can vary throughout the day, and this oscillation might be reflected in the cyclic functions of other variables like the neurotransmitter production and/or release. Dopamine (DA), adrenaline, and noradrenaline (NA) are part of the catecholamine family of neurotransmitters in which tyrosine hydroxylase (TH) is the precursor of all (Squire et al., 2008). The dopaminergic system is highly oscillating, in mice higher levels of TH mRNA production in the VTA have been shown during the early resting period (Chung et al., 2014). Similar results have been found in rats, with higher protein expression of TH at ZT6, during the light period (Webb et al., 2009). Furthermore, not only the production, but also the release of DA is highly rhythmic during the LD cycle, within the dorsal striatum (DS) of rats (Smith et al., 1992; Paulson and Robinson, 1994; Ferris et al., 2014) as well as ventral striatum of rats and mice (Castañeda et al., 2004; Hampp et al., 2008; Hood et al., 2010). The endogenous rhythmicity of DA release in the NAc has also been observed in DD conditions where higher levels are observed during the active period of rats (Castañeda et al., 2004). The rhythmic dopaminergic activity in the mesolimbic system has been reviewed previously by Webb et al. (2015). The rhythmicity of adrenaline and NA, implicated in the activation of the fight or flight behavior in response to a threatening situation (Squire et al., 2008) has also been studied. Measured from the blood of human adults, a clear rhythmic pattern is present, showing the highest levels during the active period (Scheer et al., 2009).

Serotonin (5-HT) is another important neurotransmitter within the reward system, which is produced in the raphe nuclei and projects throughout the brain. It is involved in the regulation of mood, food intake, and circadian rhythms (Versteeg et al., 2015). The levels of 5-HT and its main metabolite, 5-HIAA, have been measured during different time points. Interestingly, when measuring 5-HIAA with microdialysis in the DS and NAc of rats there is a rhythm, which is synchronized to the LD cycle where the highest levels are observed during the night. Nevertheless, when the light condition is changed to constant light (LL), the rhythms of 5-HT and 5-HIAA in both DS and NAc are ablated whereas in the DD condition the rhythm is still present in the NAc but not in the DS (Castañeda et al., 2004). A different report on the diurnal levels of 5-HT assessed the variations in rats at six different time points but found no diurnal variation of this neurotransmitter in the anterior hypothalamus or the cortex (Cagampang et al., 1993). Nevertheless, studies using microdialysis to measure 5-HT levels in the SCN of rats (Cagampang et al., 1993) and hamsters (Dudley et al., 1998) have shown that there is clear rhythmicity though there are some differences between species. Rats show higher levels during the light period whereas levels are higher during the dark period in hamsters. Despite species differences, the results point toward a rhythmic function of 5-HT that varies across brain regions.

### Rhythmic clock-gene expression

The rhythmic variation of the function and activation of the reward system can also be extended to clock-genes, which are widely expressed in these areas. In the mesolimbic system including the NAc, the PFC, DS, BNST, and amygdala the genes *Per 1–3, Clock*, and *Npas2* oscillate in a circadian manner (Harbour et al., 2013; Webb et al., 2015). The habenula, which regulates mesolimbic dopaminergic release, also shows daily variations of the *Per2* gene and protein (Zhao et al., 2015). But when the BNST, NAc and VTA are isolated and cultured for *ex-vivo* bioluminescence recordings, no rhythmicity of PER1 is observed (Abe et al., 2002). Recently, a study assessing *ex-vivo* bioluminescence showed rhythmicity of PER2 in NAc cells (Logan et al., 2015), which differs from the findings for PER1. This may indicate that different molecules from the molecular clock-machinery might persist more than others, and thus be a stronger molecular timekeeper. Using the same technique, it has been shown that in the habenula PER2 also displays robust oscillations (Guilding et al., 2010). Taken together these results indicate that the reward system has several parameters that are able to oscillate, but further research is needed to understand the physiological repercussions of these diurnal and circadian variations. The circadian function of the reward system might be altered by external factors that stimulate and modify its function. One of these possible factors is food intake, especially the ingestion of highly rewarding food that facilitates the development of obesity.

## Influence of highly caloric intake on the circadian system output

### Locomotor activity

Feeding is generally coupled to the period of arousal and (locomotor) activity. In mice and rats, the highest locomotor activity is performed at night, although some bouts of activity are also present during the day-time. This activity pattern is observed when the animals are under normo-caloric feeding conditions. However, when rats and mice are offered a high caloric diet, the normal rhythm of locomotor activity is altered showing an overall decrease in the amount of activity during the night (Mendoza et al., 2008; Sherman et al., 2012; Pendergast et al., 2013; Sun et al., 2015). Moreover, some studies in mice have found that the activity during the daytime is also disrupted, generating spares activity throughout the light period resulting in a-rhythmic activity (Pendergast et al., 2013; Branecky et al., 2015). Nevertheless, these alterations are not observed through all the studies using a highly caloric diet (Table 1). Kosaka et al., didn't find differences in the amount of activity during the day on high-fat fed vs. normal chow-fed mice under LD conditions, but the behavioral recordings (actograms) resembles the disrupted behavior reported by Pendergast et al. (2013), Kohsaka et al. (2007). In DD conditions mice fed a hyper caloric diet increased the length of their activity period compared to the normo-caloric fed mice during the first week of diet exposure (Kohsaka et al., 2007; Mendoza et al., 2008). In addition to the locomotor activity changes, similar disturbances have been observed in sleep-wake physiology recorded in mice and rats. The electroencephalogram of the animals fed with a high-fat diet showed decreased awake time as well as wake fragmentation and more rapid eye movement (REM) and non-REM sleep episodes (Jenkins et al., 2006; Guan et al., 2008; Luppi et al., 2014). This highlights that, when they are present, the effects of the diet content on general activity might evolve together with changes in sleep patterns. Data presented in this section reflect the influence of the ingestion of a high-fat diet in a pellet over the disruption of the locomotor activity. However, high caloric diets that are not only high in fat but also offer free access to sugar did not clearly find an effect on general locomotion (la Fleur et al., 2007; Oosterman et al., 2015) suggesting that not only the highly caloric content but the quality of food influences the changes observed in behavior.

**Table 1:** **Effects of *ad libitum* highly caloric diets on rhythmic behavioral outputs and clock gene expression**.

**References**	**Locomotor activity**	**Eating patterns**	**Clock genes**	**Diet**	**Species**
Branecky et al., 2015	>Amplitude	<Amplitude	*Ex-vivo* bioluminiscence Liver PER2 phase advanced	Pellet 45% kcal from fat	Male mice C57BL/6J::LUC
Sherman et al., 2012	Overall decrease	NR	qPCR Liver disrupted *Clock, Bmal1, Per2, Cry1, Cry2*	Pellet 42% kcal from fat	Male mice C57BL/6J
Sun et al., 2015	Overall decrease	NR	qPCR Liver *Clock, Bmal1* and *Per2* lost rhythm	Pellet 45% kcal from fat	Male mice C57BL/6J
Pendergast et al., 2013	Not a clear effect	<Feeding during day	PER2::Luc in the ARC complex. No change	45% kcal from fat	Male mice C57BL/6J::LUC
Mendoza et al., 2008	>Wheel running at night	NR	NR	53% kcal from fat	Male mice C57BL/6J
Kohsaka et al., 2007	No differences	<Feeding at day>Feeding at night	*Clock, Bmal1* and *Per2* qPCR, Hypothalamus No change. Fat tissue and liver > amplitude	45% kcal from fat	Male mice C57BL/6J
Guan et al., 2008	>Wake <Non REM sleep	NR	NR	59.3% kcal from fat	Male mice C57BL/6J
Jenkins et al., 2006	>Wakefulness and <NREMS	NR	NR	59.3% kcal from fat	Male mice C57BL/6J
Luppi et al., 2014	<REM and nREM sleep	NR	NR	35% fat	Male rat sprague-dawley
Oosterman et al., 2015	No differences	NR	NR	Fat and sugar choice. Average: 30.1% from fat 33.4% from sucrose	Male rat wistar
la Fleur et al., 2007	No change	NR	NR	37.4% from fat 14.8 from sucrose	Male rat wistar
Mifune et al., 2015	>Amplitude	<Kcal during day compared to chow fed group	NR	60% kcal from fat	Male rat sprague-dawly
Hariri and Thibault, 2011	NR	<Feeding at day specially from butter based pellet	NR	Pellet 65% kcal from either cannola oil or butter	Female rat sprague-dawley
Cunningham et al., 2016	>During night	>Meal events during the night	Hypothalamus qPCR in whole hypothalamic punches no effect >BMAL1 in DIO mice under DD	Pellet 60% kcal from fat, 16w	Male Mice C57BL/6J, Mice C57BL/6J::LUC
Wong et al., 2015	>At day and night with the corn oil enriched	NR	NR	40% kcal from fat in olive and corn oil enriched pellets	Female mice C57/Bl6
Jang et al., 2012	NR	<Feeding at day >Feeding at night	*Bmal1, Per2* and *Clock* in hypothalamus. No change	Pelllet 60% kcal from fat	Male mice C57BL/6N

*NR, not reported*.

### Eating patterns

When rats and mice are fed with a highly-caloric diet, some disturbances can be observed in the locomotor activity, as reviewed in the previous section. Nevertheless, the main behavioral output that is largely affected by hypercaloric consumption is the feeding behavior. The first observation is a clear over ingestion of calories and secondly, a development of a fragmented feeding pattern where rats and mice fed with a single high-fat pellet eat in small but frequent bouts instead of consolidated large meals (Kohsaka et al., 2007; Pendergast et al., 2013; Branecky et al., 2015; Mifune et al., 2015). This eating pattern resembles human snacking behavior where the caloric intake does not only rely on the three meals but in the consumption of several snacks spared in the day and night-time. Because the food intake of animals with normo caloric regimen is rhythmic and well consolidated to the active period, a snacking pattern that extends to the normal resting period might reflect an alteration in circadian rhythmicity. Moreover, this snacking pattern might be influenced by the rewarding properties of the hyper caloric foods as shown by experimental models where the rats were able to choose among different food items. In a model of free choice High-Fat High-Sugar, the rats are free to eat their most preferred food among: chow food, a dish with fat, a bottle of water and a bottle of sugar offered *ad libitum*. As expected, the animals display an overall increased caloric consumption but interestingly, the snacking behavior is mainly observed from the bottle of sugar (la Fleur et al., 2014; Oosterman et al., 2015). In another experiment with food choices, two groups of rats were given access to normal chow food, together with either canola oil (low in saturated fatty acids) or butter (high saturated fatty acids). Although both groups developed a snacking pattern, the diurnal disruption was more evident in the group fed with butter (Hariri and Thibault, 2011). These are interesting observations since the rats with free food choices barely alter their day-night consumption of chow food, a fact that might indicate that the alteration in the rhythmic feeding is influenced by the palatability of food.

### Effects of hyper caloric diets on rhythmic activity of the homeostatic neural system

When the body is challenged with a hyper caloric diet, fat stores rapidly increase, affecting the levels of circulating hormones such as leptin and corticosterone already within the first days on the diet (Buettner et al., 2007; Cano et al., 2008; la Fleur et al., 2010). Both leptin and corticosterone show diurnal rhythmity and have been described to be altered by high fat diets, mainly influencing the amplitude of the rhythm (Cha et al., 2000; Cano et al., 2008). Within the brain, NPY, 5-HT and DA are rhythmic-expressing molecules that are also affected in the obese state (Pritchet and Hajnal, 2011; Koopman et al., 2013; Gumbs et al., 2015). At the level of the clock gene expression, the hyper caloric diet largely alters clock gene expression in peripheral organs such as the liver and adipose tissue with varying effects depending on the energy content of the diet, the age and species of the animals used in the studies as well as the duration of the diet (Pendergast et al., 2013; Branecky et al., 2015; Wong et al., 2015; Cunningham et al., 2016). With regard to rhythmic expression of neuropeptides and clock-genes in the brain only few studies have focused on the effects of a diet-induced obesity (DIO). In mice, *Bmal1, Per2* and *Clock* gene expression have been assessed using qPCR in animals fed a normo-caloric and a hyper caloric diet, no differences were found within the hypothalamic area (Kohsaka et al., 2007; Jang et al., 2012). Moreover, PER2, expression, measured with *ex-vivo* bioluminescence, was not changed in the arcuate complex of mice fed with a hypocaloric diet vs. chow diet (Pendergast et al., 2013). When the hypothalamic structures were separately evaluated for *Per2* gene expression using qPCR, the high-fat diet did not exert an effect on the ARC or the DMH in DD conditions. A separate study showed, using *in situ* hybridization, decreased amplitude in expression of *Bmal1* in the SCN of DIO mice under DD conditions (Cunningham et al., 2016). In peripheral tissues such as the liver, remarkable changes caused by DIO can be observed in the clock gene expression. These changes include a PER2 phase advance in the liver of mice DIO mice, evidenced by bioluminescence (Branecky et al., 2015). Other changes include the blunted rhythmicity of Per2, Bmal1 and *clock* mRNA in the liver (Kohsaka et al., 2007; Sun et al., 2015) and white adipose tissue (Kohsaka et al., 2007). Taken together, the main findings are that the peripheral organ and peripheral circulating hormones are largely altered by a hypercaloric diet and obesity, but little to no effects of hyper caloric diets offered *ad libitum* are found on the hypothalamic clock-gene expression. One possibility is that the DIO state could be generating an uncoupling of hypothalamic and peripheral organ oscillators.

### Effects of the hyper caloric diets on rhythmic parameters in the reward system

The exposure to a highly caloric diet can change locomotor activity and feeding patterns, but little is known about the clock gene expression in the reward related areas during a diet-induced obese state. So far, no studies have focused on the effects of an *ad libitum* hyper caloric diet on the clock-gene expression or other oscillatory properties of the reward system. Nevertheless, the influence of the pleasurable food on the behavioral rhythmic outputs has been evidenced with different experimental paradigms. During a palatable scheduled feeding experiments, a palatable treat is given daily at the same time during several days to animals fed with regular chow *ad libitum*. In this way, the rewarding properties of the food are dissociated from the metabolic needs. The behavioral outcomes from these studies done in both mice and rats, show that animals can entrain their behavior, developing an anticipatory general activity previous to the food (Mendoza et al., 2005; Angeles-Castellanos, 2008; Hsu et al., 2010; Gallardo et al., 2012; Merkestein et al., 2012). During this palatable food anticipation, the NAc, PFC and LH showed an increase in *Per1* (Angeles-Castellanos, 2008). In a similar study, *Per2* was unchanged in the BNST and amygdala of rats (Verwey et al., 2007), suggesting that the effects of palatable food under a scheduled feeding regimen depend on the clock-gene and brain area. The experiments discussed in this section used a palatable treat, given daily in a small amount that did not result in body weight gain and thus conclusions can only be drawn about palatable intake effects on the rhythmic clock-gene expression in the reward system and not about DIO effects.

## Conclusion

**Figure 1:**
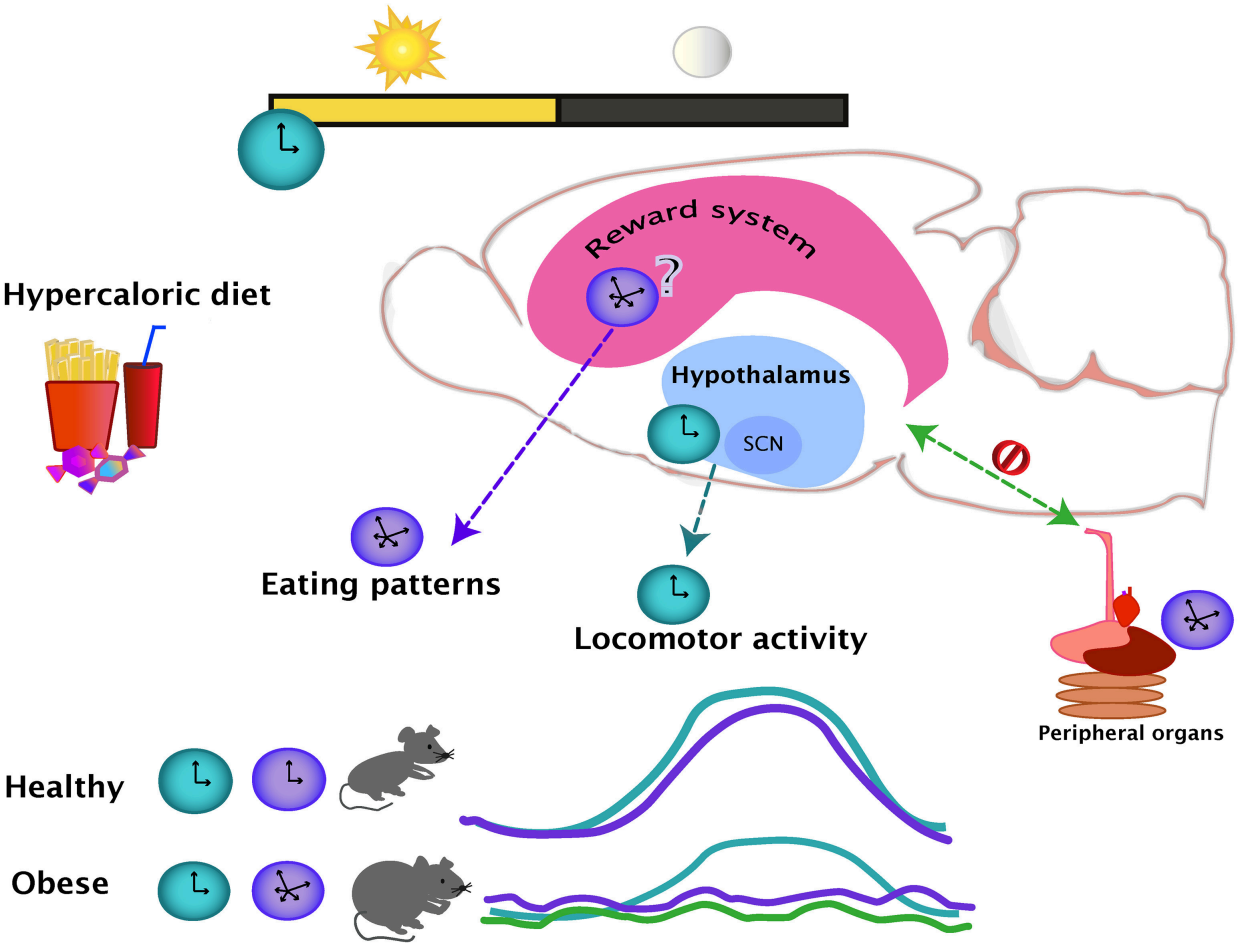
**The day-night cycles set the regular oscillations of eating (purple line) and locomotor activity (blue line), which are coupled during a healthy state**. Intake of hypercaloric diets, leading in obesity, disrupts the eating daily patterns, producing small but frequent bouts of ingestion even during the normal resting period. The locomotor activity and eating pattern rhythms are uncoupled in an obese state. The effects of a hypercaloric diet over the rhythmicity of the reward system are unknown but as the evidence suggest that the rhythmicity in the hypothalamus is mainly unaffected (blue dotted line), the reward system might be influencing the disturbances of the daily eating patterns (purple dotted line). In the diet-induced obese state, the rhythmicity of the peripheral organs are altered (green line), causing an internal desynchrony of central and peripheral oscillators (green dotted line).

The effects of obesity in general physiology have been widely studied and a large amount of knowledge has been gathered about the changes within the brain produced by hyper caloric diets. Nevertheless, the changes in circadian outputs like locomotor activity and eating patterns are not reported in most of these studies. From the studies discussed in the present review, it appears that the access to a hyper caloric regime does not alter general locomotor activity to the same extent as the food intake rhythmicity. One possibility for this might be that the rhythmic locomotor output is more resistant to change due to the lack of effects in the SCN and in other hypothalamic areas, while the eating patterns guided by the food palatability might be changing together with the changes in the brain reward system (Figure 1). No conclusion can be drawn at this point due to the fact that the effects of a hyper caloric diet on the rhythmic brain parameters is inconclusive for the hypothalamus and non-existent for the nuclei from the reward system. At level of the brain, studies are region and system-specific, and therefore, the findings might differ due to varying methodological approaches. Nevertheless, a study looking at the broad spectrum of metabolomics and genomics supports the observations of altered gene expression in obesity (Eckel-Mahan et al., 2013). Showing how the relationship of the reward and metabolic systems integrate and intercommunicate with circadian function might be a step to gaining a better understand of the causes and consequences of obesity.

## Author contributions

AB, JM, and Sl structured, designed, and wrote the content of the review. AG revised the content and edited the writing of the work.

## Funding

AB was supported by a doctoral fellowship from the Neurotime Erasmus Mundus Program; JM was supported by the Agence National de la Recherche (ANR-14-CE13-0002-01 ADDiCLOCK JCJC to JM), the Centre National de la Recherche Scientifique (JM), and the Institut Danone France-Fondation pour la Recherche Médicale Consortium (JM); AG and Sl were supported by a NWO-VICI grant (project No. 016.160.617)

### Conflict of interest statement

The authors declare that the research was conducted in the absence of any commercial or financial relationships that could be construed as a potential conflict of interest.
